# Application of blood flow restriction in hypoxic environment augments muscle deoxygenation without compromising repeated sprint exercise performance

**DOI:** 10.1113/EP091032

**Published:** 2023-03-19

**Authors:** Anjie Wang, R. Matthew Brothers, Chansol Hurr

**Affiliations:** ^1^ Integrative Exercise Physiology Laboratory, Department of Physical Education, College of Education Jeonbuk National University Jeonju South Korea; ^2^ Integrative Vascular Physiology Laboratory, Department of Kinesiology College of Nursing and Health Innovation University of Texas at Arlington Arlington TX USA

**Keywords:** blood flow restriction, muscle oxygenation saturation, neuromuscular activation, repeated spring exercise, systemic hypoxia

## Abstract

Repeated sprint exercise (RSE) is a popular training modality for a wide variety of athletic activities. The purpose of this study was to assess the combined effects of systemic hypoxia and blood flow restriction (BFR) on muscle deoxygenation and RSE performance. Twelve healthy young men performed a standard RSE training modality (five sets of 10 s maximal sprint with a 60 s rest) under four different conditions: (1) normoxic control (NC), normoxia (N, 20.9%) + control BFR (C, 0 mmHg); (2) normoxic BFR (NB), normoxia (N, 20.9%) + BFR (B, 140 mmHg); (3) hypoxic control (HC), hypoxia (H, 13.7%) + control BFR (C, 0 mmHg); and (4) hypoxic BFR (HB): hypoxia (H, 13.7%) + BFR (B, 140 mmHg). BFR was only administered during the rest period of the respective RSE trials. In the local exercising muscles, muscle oxygen saturation ($\mathrm{Sm}O_{2}$) and neuromuscular activity were measured using near‐infrared spectroscopy and surface electromyography, respectively. SmO_2_ was lower in systemic hypoxia conditions relative to normoxia conditions (*P* < 0.05). A rther decrease in SmO_2_ was observed in HB relative to HC (Set 1: HC 70.0 ± 17.5 vs. HB 57.4 ± 11.3%, *P* = 0.001; Set 4: HC 67.5 ± 14.6 vs. HB 57.0 ± 12.0%, *P* = 0.013; Set 5: HC 61.0 ± 15.3 vs. HB 47.7 ± 11.9%, *P* < 0.001). No differences in RSE performance were observed between any of the conditions (*P* > 0.05). Interestingly, an elevated neuromuscular activity was seen in response to the BFR, particularly during conditions of systemic hypoxia (*P* < 0.05). Thus, RSE with BFR administered during rest periods under systemic hypoxia led to severe local hypoxia without compromising training workload.

## Abstract (highlights)

- **What is the central question of this study?**
  Does applying blood flow restriction during the rest periods of repeated sprint exercise in a hypoxic environment lead to greater local hypoxia within exercising muscles without compromising training workload?
- **What is the main finding and its importance?**
  Repeated sprint exercise with blood flow restriction administered during rest periods under systemic hypoxia led to severe local hypoxia within the exercised muscles without a reduction in power output. The maintained power output might be due to elevated neuromuscular activation. Accordingly, the proposed repeated sprint exercise in the current study may be an effective training modality.

## 1 INTRODUCTION

Repeated sprint exercise (RSE) is a training modality widely used in many sports, such as football (soccer) and rugby (Bishop et al., 2011; Spencer et al., 2005). RSE training is associated with elevated metabolite accumulation such as hydrogen ion (H^+^) and inorganic phosphate (P_i_) in the tissue of the exercised muscles (Bishop et al., 2011). Accordingly, changes in the haemodynamics of the exercising muscle tissue are closely related to metabolite production and clearance, such that a reduction in blood flow directed towards the working muscle elicits greater stimuli to exercising muscle tissue (Mitchell et al., 2019).

Blood flow restriction (BFR) is an emerging training modality that enhances muscle strength and endurance (Schoenfeld, 2013). BFR occludes venous return while simultaneously partially reducing arterial inflow, resulting in localized hypoxia and increased metabolite stress (i.e., phosphocreatine depletion, accumulation of P_i_, H^+^ and lactate) in the occluded muscle tissue (Pearson & Hussain, 2015; Schoenfeld, 2013). Although BFR triggers local hypoxia in muscle tissues, power output is attenuated when BFR is applied continuously during RSE training (sprint + rest) (Willis, Borrani et al., 2019). Other researchers have likewise reported that BFR lowers the number of sprints that can be performed (Valenzuela et al., 2019) as well as sprint distance completed (Peyrard et al., 2019). For well‐trained athletes, however, a power output during RSE (i.e., training workload) needs to be maximized to ensure the best training benefits (Scott et al., 2016), prompting researchers to examine BFR. Torma et al. (2021) found that when administered during rest periods, BFR is an effective training strategy that enhances angiogenesis‐associated gene expression, mitochondrial biogenesis, muscle repair and hypertrophy. More recently Kojima et al. (2021) revealed that application of BFR during RSE rest periods provides greater hypoxic stimuli in working muscles without reducing subsequent power output.

Compensatory systemic vasodilatation occurs in a hypoxic environment, in an effort to ensure that the muscle tissue receives a constant oxygen supply (Casey & Joyner, 2012; Wilkins et al., 2008). Despite differing intrinsic mechanisms, systemic hypoxia and BFR each leads to a local hypoxic environment in the exercising muscles (Willis, Peyrard et al., 2019). While some studies showed a potent local hypoxia within exercising muscles in response to BFR application under systemic hypoxia, power output during RSE training was reduced in the combined condition (hypoxia + BFR) possibly due to the continuous application of BFR during RSE (sprint + rest) (Willis, Borrani et al., 2019, Willis, Peyrard et al., 2019).

Accordingly, the aim of the present study is to fill this gap and examine the impact of combined systemic hypoxia and BFR on RSE training. To minimize the effect on exercise workload, BFR was only administered during RSE rest periods. In addition, RSE leads to the rapid development of neuromuscular fatigue, as demonstrated by a reduction in the central drive to the active musculature and an impaired muscle activation (Mendez‐Villanueva et al., 2012, 2008). Several studies have demonstrated that neuromuscular activity is significantly augmented during exercise under conditions of limited blood flow as compensatory mechanisms to maintain power output (Girard et al., 2019; Husmann et al., 2018). To determine muscle activation and recruitment strategy during sprints of RSE under normoxia and hypoxia, neuromuscular activity was assessed by surface electromyography (EMG) in the current study. We hypothesized that the combined condition of systemic hypoxia + BFR would induce a greater magnitude of muscle deoxygenation and neuromuscular activation than either intervention administered in isolation. Furthermore, it is hypothesized that these combined conditions would not result in a reduction in RSE performance.

## 2 METHODS

### 2.1 Ethical approval

All experimental procedures were approved by the Jeonbuk National University Ethics Committee (Ref. No.: JBNU 2022‐07‐019‐001) and conformed to the standard set by the *Declaration of Helsinki* (2013), except for registration in a database. All subjects were verbally informed of the risks and discomforts associated with experimental trials, and written informed consent was obtained from all participants prior to their participation.

### 2.2 Participants

Twelve young and healthy men (age: 26.4 ± 3.6 years, height: 1.79 ± 0.05 m, body mass 75.0 ± 8.8 kg, body fat: 11.9 ± 2.5%) who regularly participated in a variety of exercises including running, cycling, swimming and resistance training (>1 h/day at least on 3 days/week) participated in the present study. Participants were asked to maintain their diet during the testing period and to refrain from strenuous physical activity, alcohol and caffeine consumption during the 24 h prior to all study visits. All participants lived near sea‐level during the test, and reported no exposure to an altitude of ≥3000 m in the 3 months that preceded the study. No participant had a history of severe acute mountain sickness.

### 2.3 Experimental protocol

**FIGURE 1:**
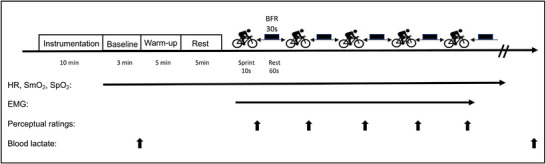
Experimental protocols. BFR, blood flow restriction; EMG, electromyography; HR, heart rate; SmO_2_, muscle oxygen saturation; SpO_2_, pulse oxygen saturation.

A schematic representation of the experimental procedure is presented in Figure 1. The current study was conducted using a randomized, crossover design, in which participants were blind to the environmental conditions (i.e., normobaric hypoxia or normoxia). For logistical reasons, BFR condition could not be blinded as participants could easily recognize the pressure of the cuff associated with BFR administration. Participants came to the laboratory for a total of five visits, which included one familiarization and four data‐collections visits. During each data‐collection visit, each participant performed a RSE (10 s maximal sprint with 60 s rest in each set, for a total of five sets) under four different conditions: (1) normoxic control condition (NC), normoxic inspired oxygen (N, 20.9%) + 0 mmHg of BFR occlusion (C); (2) normoxic BFR condition (NB), normoxic inspired oxygen (N, 20.9%) + 140 mmHg of BFR occlusion (B); (3) hypoxic control condition (HC), hypoxic inspired oxygen (H, 13.7%) + 0 mmHg of BFR occlusion (C); and (4) hypoxic BFR condition (HB), hypoxic inspired oxygen (H, 13.7%) + 140 mmHg of BFR occlusion (B). These four visits were completed in a randomized order with at least 72 h separation between each visit. With each participant, all visits occurred at the same time of day (±1 h). Muscle oxygen saturation (SmO_2_) and total haemoglobin (Total‐Hb) were measured using near‐infrared spectroscopy (NIRS), and neuromuscular activity was assessed by surface EMG. Other basic physiological indexes, including pulse oxygen saturation (SpO_2_), heart rate (HR), blood lactate and perceived discomfort (muscle and breathing), were assessed as well (Figure 1).

Upon arrival in the laboratory, each participant entered a climate chamber and rested for 10 min for environmental acclimation (Goto et al., 2018). During this period, participants were instrumented with NIRS, wireless EMG sensors, SpO_2_ and BFR cuffs (see below for instrumentation details). Participants then sat for another 3 min to obtain baseline measurements and resting blood lactate concentrations. Following the acquisition of baseline measurement, participants performed a warm‐up exercise (5 min pedalling at 60 rpm with 1.5 kg load and 2 × 3 s maximal pedalling with a load equivalent to 5% of their body mass), followed first by a 5‐min passive recovery then a standardized RSE (5 × 10 s maximal pedalling exercise with the load of equivalent to 7.5% of body mass, separated by a 60‐s rest between sprints) using a cycle ergometer (Monark 894e, Vansbro, Sweden) (Barber et al., 2013; Kojima et al., 2021). During a 60 s rest between sprints, participants remained on the ergometer and maintained an upright posture by dropping the right leg while sitting on the bike. During the BFR trial (NB and HB), the cuffs were inflated to 140 mmHg for 30 s immediately after each sprint (inflation and deflation of the cuff took ∼15 s, respectively). In trials with BFR of 0 mmHg (NC and HC), the cuffs remained deflated while participants were asked to maintain an identical posture to that used in trials in which the BFR was set at 140 mmHg (NB and HB). Variables in RSE performance (peak power, mean power, minimum power and power drop) were normalized to body mass and total work was analysed with absolute data (kJ).

#### 2.3.1 Climate chamber

All sessions were completed in a climate chamber (chamber size: 7 m length × 4 m width × 3 m height) and the hypoxic environment was controlled using a hypoxic generator (JAY‐60H, Longfian, Baoding, China) at an elevation of 400 m above sea level (Jeollabuk‐do, Korea). Environmental temperature in the chamber was maintained at a constant ∼21°C with a humidity of ∼50%. The targeted fraction of the inspired oxygen in the atmosphere (FiO_2_) levels for the normoxic and hypoxic conditions were set at 20.9 (∼400 m above sea level) and 13.7% (∼3000 m above sea level), respectively, and FiO_2_ levels during the procedure were continuously monitored by a wireless oxygen gas analyser (AR8100, Smart Sensor, Dongwan, China).

#### 2.3.2 Blood flow restriction

Automatically inflatable BFR cuffs (band width: 5 cm, KAATSU C3, Sato Sports Plaza, Tokyo, Japan) were placed as high up as possible on the proximal portion of each thigh consistent with best practices (Taylor et al., 2016; Weatherholt et al., 2019). The occlusion pressure of 140 mmHg was used in the current study as the pressure of 140 mmHg restricts resting blood flow to the popliteal artery by ∼76%, which meets the recommended arterial occlusion pressure (AOP) for suitable ‘minimum’ BFR pressure (60∼80% AOP) (Ilett et al., 2019).

### 2.4 Measurements

#### 2.4.1 Near‐infrared spectroscopy

Muscle oxygenation profiles of the right vastus lateralis (VL) were assessed using a portable NIRS apparatus (Idiag Moxy, Idiag, Fehraltorf, Switzerland). The probe was placed on the right thigh, at one‐third of the way between the great trochanter and knee joint (VL of right legs, all dominant legs) and wrapped with a bandage to protect it from light and sweat. A marker was used to mark the position of the device and ensure that its placement was reproduced during subsequent visits. The NIRS device uses four wavelengths of light (680, 720, 760 and 800 nm) and the sensor contains two detectors at distances of 12.5 mm (for superficial) and 25.0 mm distances (for deeper tissue) based on the source–detector system (Vrana et al., 2018).

All NIRS signals were acquired at a default sampling rate which samples the four wavelengths over 80 cycles for an averaged output every 2 s and averaged out for data analysis. For analysis, a bin‐averaging strategy was used to determine the SmO_2_ and total‐Hb signal at the time points of interest (Inglis et al., 2019). Baseline values were established and averaged 1 min before the warm‐up period, during which participants sat for 3 min. During the RSE, the SmO_2_ and total‐Hb values were recorded, and an average was taken for the 10 s sprints and 30 s rest periods (between second 15 and 45 s of the 60 s). According to the previous work, a moving average with a window of 2 s was applied as a filtering method for data analysis (Rodriguez et al., 2018). Also, normalization for the amplitude of SmO_2_ and total‐Hb were done on an individual basis for each session with the amplitude from baseline representing a 100% amplitude (Smith & Billaut, 2010; Subudhi et al., 2007).

#### 2.4.2 Electromyography

Surface EMG signals were recorded during the sprint exercise to assess neuromuscular activity. A wireless surface EMG system with a sampling frequency of 2000 Hz (Trigno Wireless EMG System, Delsys Inc., Natick, MA, USA) was used. The sensor was placed one‐third of the distance between the great trochanter to knee joint (VL of left legs, all non‐dominant legs). Before electrode placement, skin was lightly shaved and washed to remove surface debris and oil, and electrodes were secured with an elastic adhesive bandage to reduce movement during exercise. The position of the EMG electrodes was marked with a pen (consistent with NIRS assessment guidelines) to ensure consistent placement across visits. The myoelectric signals were processed off‐line with analysis software (Delsys EMG Works Analysis 4.2.0, Delsys Inc.). The filtering range was 10–500 Hz to reduce the noise in the low‐frequency region and eliminate motion artifacts in the high‐frequency region. Muscle activity was determined by measuring the mean value of the root mean square (RMS) and mean frequency (MF) associated with each sprint. Using the same approach described in previous studies (Billaut et al., 2013; Smith & Billaut, 2010), RMS data for Sets 2–4 of the RSE were normalized to the first sprint (expressed as a percentage of signal amplitude in reference to the Set 1).

#### 2.4.3 HR and SpO_2_

HR was recorded continuously via a Polar transmitter‐receiver (OH1, Polar Electro Oy, Kempele, Finland) and averaged across every sprint and rest period. SpO_2_ was monitored after each sprint and 5 s before each began using non‐invasive pulse oximetry (YX306, Yuwell Mecical Inc., Jiang Su, China). All participants were monitored on the index finger of their right hand (in each case, the dominant hand) in a manner that did not allow participants to view any data (Girard et al., 2019).

#### 2.4.4 Perceived discomfort scales

Perceived muscle discomfort and difficulty breathing were immediately assessed after each sprint using the Borg CR10 scales which range from 0 (no effort at all) to 10 (maximum effort) (Borg, 1982). During the familiarization, participants were instructed that these ‘perceived discomfort’ scales would be used to evaluate their subjective perception of (1) thigh muscle fatigue (‘How uncomfortable do your legs feel?’) and (2) difficulty breathing (‘How uncomfortable does it feel to breath?’) (Girard et al., 2019). After the RSE test was completed in its entirety, participants were instructed to reconfirm their perceptions.

#### 2.4.5 Blood lactate concentration

Blood samples were collected from the fingertip to evaluate blood lactate concentration prior to warm‐up and 5 min after the last sprint of the RSE. Blood lactate concentrations were measured immediately after collection using a lactate analyser (Accutrend Lactate, Roche Diagnostics GmbH, Mannheim, Germany).

### 2.5 Statistical analyses

Data are expressed as means ± SD. A two‐way repeated measures ANOVA was used to determine the main effects of condition, time, and condition × time, followed by a Tukey's *post hoc* analysis. Statistical significance was determined at *P* < 0.05. Consistent with an a priori sample‐size calculation (G*Power 3.1) made in previous studies (Girard et al., 2019; Kojima et al., 2021), 12 participants per condition were required to yield the targeted statistical power of β = 0.90 at α = 0.05 for the repeated sprint tests (actual power = 0.97 at *n* = 12).

Partial eta‐squared (η^2^) was calculated to estimate the effect size of the two‐way ANOVA (main effects and interaction), in which values of 0.01, 0.06 and above 0.14 represent small, medium and large effects, respectively. In addition, Cohen conventions for effect size (ES) were used when a *P* < 0.05 was detected between conditions, in which case an ES equal to 0.2, 0.5 and 0.8 was considered a small, medium and large effect, respectively. All statistical calculations were performed using Prism 8.3 software (GraphPad Software, San Diego, CA, USA).

## 3 RESULTS

Changes in power output are presented in Table 1. Peak power, mean power, minimum power output and total work all significantly decreased between the second to fifth set of sprints across all conditions (*P* < 0.001 for all comparisons; η^2^ = 0.949, 0.967, 0.903 and 0.965, respectively). However, no significant main effect of condition and interactions were observed during any of the tests of the examined variables (*P* > 0.05 for all comparisons). Furthermore, *post hoc* comparisons revealed no differences in any indexes of RSE performance between conditions (*P* > 0.05 for all comparisons).

**TABLE 1:** Power output during repeated sprint exercise.

					Main effects		
	NC	NB	HC	HB	Time	Condition	Interaction
Peak power (W/kg)							
Set 1	10.56 ± 0.83	10.69 ± 1.17	10.44 ± 1.13	10.36 ± 1.26	*P* < 0.001	NS	NS
Set 2	8.47 ± 1.12^*^	8.66 ± 1.36^*^	8.73 ± 1.35^*^	8.38 ± 1.33^*^			
Set 3	7.22 ± 1.23^*#^	7.26 ± 1.24^*#^	7.35 ± 1.14^*#^	7.24 ± 1.49^*#^			
Set 4	6.19 ± 0.90^*#^	6.29 ± 1.11^*#^	6.52 ± 1.44^*#^	6.21 ± 1.29^*#^			
Set 5	5.68 ± 1.05^*^	5.84 ± 1.21^*#^	6.13 ± 1.40^*#^	5.86 ± 1.20^*^			
Average power (W/kg)							
Set 1	9.04 ± 0.73	9.37 ± 1.03	9.05 ± 0.96	9.05 ± 0.92	*P* < 0.001	NS	NS
Set 2	7.54 ± 0.82^*^	7.63 ± 1.14^*^	7.65 ± 0.95^*^	7.45 ± 1.07^*^			
Set 3	± 0.90^*#^	6.42 ± 1.06^*#^	6.48 ± 1.07^*#^	6.31 ± 1.19^*#^			
Set 4	5.46 ± 0.76^*#^	5.54 ± 1.10^*#^	5.66 ± 1.22^*#^	5.48 ± 1.09^*#^			
Set 5	5.02 ± 0.97^*^	5.12 ± 1.15^*#^	5.34 ± 1.15^*#^	4.96 ± 0.99^*#^			
Total work (kJ)							
Set 1	6.89 ± 1.17	7.09 ± 1.28	6.95 ± 0.91	6.80 ± 1.26	*P* < 0.001	NS	NS
Set 2	5.79 ± 0.99^*^	5.90 ± 1.12^*^	5.96 ± 0.92^*^	5.62 ± 1.26^*^			
Set 3	4.74 ± 0.86^*#^	4.96 ± 1.02^*#^	5.06 ± 1.02^*#^	4.78 ± 1.26^*#^			
Set 4	4.21 ± 0.82^*#^	4.33 ± 0.96^*#^	4.40 ± 1.21^*#^	4.11 ± 1.12^*#^			
Set 5	3.89 ± 0.81^*^	4.01 ± 1.01^*#^	4.12 ± 1.09^*#^	3.81 ± 0.98^*#^			
Minimum power (W/kg)							
Set 1	7.68 ± 1.34	8.00 ± 1.00	7.61 ± 1.04	7.44 ± 0.81	*P* < 0.001	NS	NS
Set 2	6.44 ± 1.16^*^	6.31 ± 1.32^*^	6.50 ± 1.05^*^	6.12 ± 1.27^*^			
Set 3	4.87 ± 1.15^*#^	5.08 ± 1.17^*#^	4.95 ± 1.34^*#^	5.03 ± 1.15^*#^			
Set 4	4.21 ± 1.17^*^	4.31 ± 1.04^*#^	4.32 ± 1.12^*^	4.32 ± 0.93^*^			
Set 5	4.21 ± 1.08^*^	3.86 ± 1.20^*^	3.95 ± 0.01^*^	3.71 ± 0.82^*^			
Power drop (W/kg)							
Set 1	2.85 ± 0.98	2.68 ± 0.69	2.82 ± 0.65	2.92 ± 1.39	*P* < 0.001	NS	NS
Set 2	2.13 ± 0.95	2.35 ± 1.15	2.23 ± 1.12	2.26 ± 0.90			
Set 3	2.91 ± 0.47	2.18 ± 0.61	2.40 ± 1.12	2.21 ± 0.74			
Set 4	2.02 ± 0.84^*^	1.98 ± 0.59	2.20 ± 0.80	1.89 ± 0.65			
Set 5	1.64 ± 0.43^*^	1.97 ± 0.52^*^	2.17 ± 0.90	2.15 ± 0.78			

Data are means ± SD. ^*^ *P* < 0.05 versus Set 1. ^#^ *P* < 0.05 versus previous set. No significant differences were found between conditions for all analyses. HB, hypoxia with BFR; HC, hypoxia with control BFR; NB, normoxia with BFR; NC, normoxia with control BFR.

**FIGURE 2:**
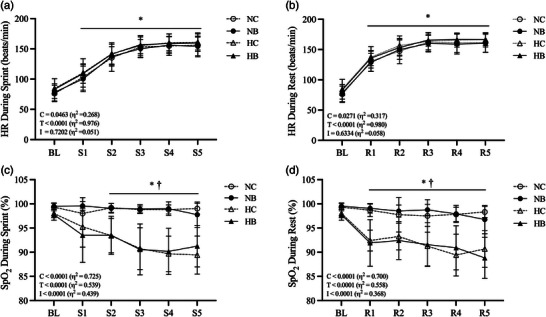
Heart rate (HR) and pulse oxygen saturation (SpO_2_). HR during sprint (a) and rest (b) and SpO_2_ during sprint (c) and rest (d) are shown. Data are means ± SD. ^*^ *P* < 0.05 versus BL. ^†^ *P* < 0.05 between normoxic and hypoxic conditions. BL, baseline; HB, hypoxia with BFR; HC, hypoxia with control BFR; NB, normoxia with BFR; NC, normoxia with control BFR.

Changes in HR and SpO_2_ levels are shown in Figure 2. Significant main effects of condition and time were detected in HR during sprint exercise (*P* = 0.046 and *P* < 0.001, respectively, η^2^ = 0.268 and 0.976, respectively) (Figure 2a) and rest periods (*P* = 0.027 and *P* < 0.001, respectively, η^2^ = 0.317 and 0.980, respectively) (Figure 2b). However, there was no significant interaction (condition × time) during sprint (*P* = 0.720) and rest periods (*P* = 0.633).

Significant main effects of condition and time, as well as an interaction, were observed in SpO_2_ levels during sprint exercise (*P* < 0.001 for all; η^2^ 0.725, 0.539 and 0.439, respectively) (Figure 2c) and rest periods (*P* < 0.001 for all; η^2^ = 0.700, 0.558 and 0.368, respectively) (Figure 2d). As expected, SpO_2_ levels in hypoxic conditions were lower relative to those in normoxic conditions during the sprint and rest periods (*P* < 0.05 for all comparisons) (Figure 2c,d), while no differences in SpO_2_ level were observed between each environmental condition during sprint and rest periods (*P* > 0.05 for all comparisons).

**FIGURE 3:**
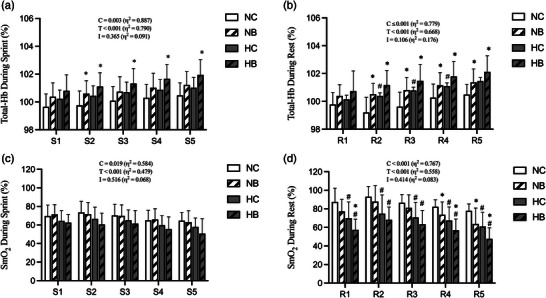
Near‐infrared spectroscopy (NIRS). Total haemoglobin (Total‐Hb) during sprint (a) and rest (b) as well as muscle oxygen saturation (SmO_2_) during sprint (c) and rest (d) are presented. Data are means ± SD. ^*^ *P* < 0.05 versus control BRF within each environmental condition. ^#^ *P* < 0.05 versus normoxia within each BFR condition. HB, hypoxia with BFR; HC, hypoxia with control BFR; NB, normoxia with BFR; NC, normoxia with control BFR.

Changes in total‐Hb and SmO_2_ levels are presented in Figure 3. Statistical analysis revealed the significant main effect of condition and time on Total‐Hb during sprints (*P* = 0.003 and *P* < 0.001, respectively; η^2^ = 0.887 and 0.790, respectively) (Figure 3a) and rest periods (*P* < 0.001 for both; η^2^ = 0.779 and 0.668, respectively) (Figure 3b). There were no interactions (condition × time) for Total‐Hb during sprint (*P* = 0.365) or rest periods (*P* = 0.106). There was a trend towards higher Total‐Hb in BFR conditions than in control BFR conditions within each environmental condition during sprints (S2: NC 99.8 ± 1.0 vs. NB 100.6 ± 0.9%, *P* = 0.042, ES = 0.84, HC 100.4 ± 0.7 vs. HB 101.1 ± 1.0%, *P* = 0.006, ES = 0.81; S3: HC 100.7 ± 0.7 vs. HB 101.3 ± 1.1%, *P* = 0.019, ES = 0.65; S4: HC 100.9 ± 0.7 vs. HB 101.6 ± 1.0%, *P* = 0.011, ES = 0.81; S5: HC 101.0 ± 0.7 vs. HB 101.9 ± 1.1%, *P* = 0.006, ES = 0.98) and rest periods (R2: NC 99.2 ± 1.4 vs. NB 100.5 ± 1.5%, *P* = 0.015, ES = 0.91, HC 100.4 ± 0.8 vs. HB 101.2 ± 1.1%, *P* = 0.035, ES = 0.83; R3: NC 99.6 ± 1.3 vs. NB 100.8 ± 1.3%, *P* = 0.046, ES = 0.92, HC 100.8 ± 0.8 vs. HB 101.5 ± 1.2%, *P* = 0.033, ES = 0.69; R4: NC 100.3 ± 0.9 vs. NB 101.2 ± 1.1%, *P* = 0.041, ES = 0.87, HC 101.1 ± 0.8 vs. HB 101.8 ± 1.1%, *P* = 0.016, ES = 0.73; R5: NC 100.5 ± 1.1 vs. NB 101.4 ± 1.0%, *P* = 0.050, ES = 0.86, HC 101.5 ± 0.9 vs. HB 102.1 ± 1.2%, *P* = 0.038, ES = 0.57) (Figure 3a,b). Also, total‐Hb tended to be higher in rest periods of hypoxic environment than normoxic environment (R2: NC 99.2 ± 1.4 vs. HC 100.4 ± 0.8%, *P* = 0.010, ES = 0.97; R3: NC 99.6 ± 1.3 vs. HC 100.8 ± 0.8%, *P* = 0.013, ES = 0.98; R4: NC 100.3 ± 0.9 vs. HC 101.1 ± 0.8%, *P* = 0.039, ES = 0.88) (Figure 3b).

There were significant main effects of condition and time on SmO_2_ levels during sprints (*P* = 0.019 and *P* < 0.001, respectively; η^2^ = 0.584 and 0.479, respectively) and rest periods (*P* < 0.001 for both; η^2^ = 0.767 and 0.558, respectively). No significant interactions (condition × time) on SmO_2_ during sprint (*P* = 0.516) and rest periods (*P* = 0.414) were detected. During rest periods, SmO_2_ was lower in the hypoxic environment than normoxic environment (R1: NC 87.5 ± 14.7 vs. HC 70.0 ± 17.5%, *P* = 0.014, ES = 0.47, NB 77.4 ± 12.7 vs. HB 57.4 ± 11.3%, *P* = 0.004, ES = 0.64; R2: NC 93.1 ± 11.0 vs. HC 74.9 ± 20.2%, *P* = 0.017, ES = 0.49, NB 87.8 ± 17.2 vs. HB 68.3 ± 13.6%, *P* = 0.001, ES = 0.53; R3: NC 86.6 ± 8.9 vs. HC 70.8 ± 16.3%, *P* = 0.018, ES = 0.52, NB 81.2 ± 14.0 vs. HB 63.5 ± 14.9%, *P* = 0.002, ES = 0.52; R4: NC 82.3 ± 7.5 vs. HC 67.5 ± 14.6%, *P* = 0.006, ES = 0.54, NB 73.6 ± 13.8 vs. HB 57.0 ± 12.0%, *P* = 0.004, ES = 0.54; R5: NC 77.8 ± 7.4 vs. HC 61.0 ± 15.3%, *P* = 0.004, ES = 0.57, NB 63.7 ± 17.1 vs. HB 47.7 ± 11.9%, *P* = 0.035, ES = 0.48). SmO_2_ in HB was even lower than in HC during R1, R4 and R5 (R1: HC 70.0 ± 17.5 vs. HB 57.4 ± 11.3%, *P* = 0.001, ES = 0.63; R4: HC 67.5 ± 14.6 vs. HB 57.0 ± 12.0%, *P* = 0.013, ES = 0.45; R5: HC 61.0 ± 15.3 vs. HB 47.7 ± 11.9%, *P* < 0.001, ES = 0.72) while no differences in SmO_2_ levels were observed between the HC and HB conditions during sprints (Figure 3c,d).

**FIGURE 4:**
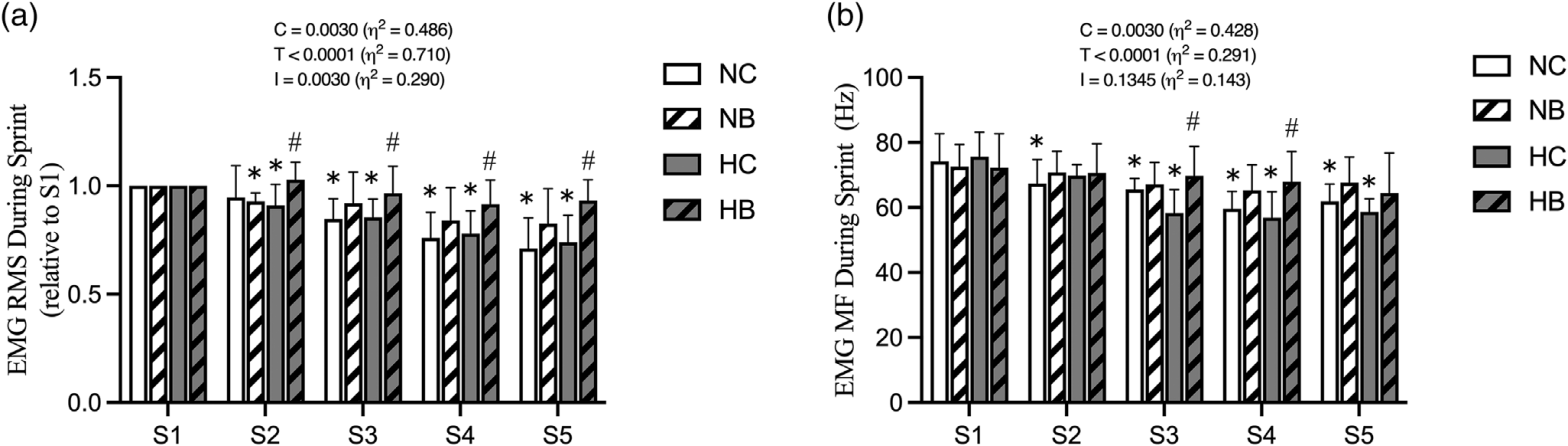
Electromyography (EMG). EMG root mean square (RMS) (a) and mean frequency (MF) (b) during sprint are shown. Data are mean ± SD. ^*^ *P* < 0.05 versus S1, #*P* < 0.05 versus control BFR within each environmental condition. HB, hypoxia with BFR; HC, hypoxia with control BFR; NB, normoxia with BFR; NC, normoxia with control BFR.

Changes in RMS and MF levels during sprints are shown in Figure 4. Significant interaction (condition × time) and main effect for time and condition were detected in the RMS data (*P* = 0.003, *P* < 0.001 and *P* = 0.003, respectively; η^2^ = 0.290, 0.710 and 0.486, respectively). RMS in HB was higher than in HC during S2, S3, S4 and S5 (S2: HC 0.91 ± 0.10 vs. HB 1.03 ± 0.08 relative to S1, *P* < 0.001, ES = 0.84; S3: HC 0.85 ± 0.09 vs. HB 0.97 ± 0.12 relative to S1, *P* = 0.004, ES = 0.79; S4: HC 0.78 ± 0.11 vs. HB 0.92 ± 0.11 relative to S1, *P* = 0.001, ES = 0.71; S5: HC 0.74 ± 0.13 vs. HB 0.93 ± 0.10 relative to S1, *P* < 0.001, ES = 0.87) (Figure 4a).

As to MF levels, two‐way ANOVA revealed a significant main effect by time and condition (*P* < 0.001 and *P* = 0.003, respectively; η^2^ = 0.291 and 0.428, respectively). No significant interactions (condition × time) for MF (*P* = 0.135) were detected. During S3 and S4, MF was higher in HB than HC (S3: HC 58.27 ± 7.25 vs. HB 69.72 ± 9.11 Hz, *P* = 0.004, ES = 0.541; S4: HC 56.88 ± 7.95 vs. HB 67.93 ± 9.29 Hz, *P* = 0.011, ES = 0.461) (Figure 4b).

Table 2 highlights the significant main effect that time exerts on muscle discomfort and difficulty breathing (*P* < 0.001 for all comparisons; η^2^ = 0.923 and 0.932, respectively). No significant main effect for condition and interaction were detected (*P* = 0.088 and *P* = 0.350, respectively; η^2^ = 0.222 and 0.094, respectively). In Table 3, blood lactate concentrations were significantly higher post‐exercise than pre‐exercise (*P* < 0.001; η^2^ = 0.963), but no significant main effects for condition and interaction were found (*P* = 0.475 and *P* = 0.822, respectively; η^2^ = 0.043 and 0.020, respectively). Also, no differences in perceived pain or blood lactate concentration were detected between conditions (*P* > 0.05 for all comparisons).

**TABLE 2:** Perceived muscle fatigue and breathing difficulty immediately following the completion of each sprint.

	NC	NB	HC	HB	Main effects		Interaction
					Time	Condition	
Muscle discomfort							
Set 1	5.2 ± 1.4	4.6 ± 1.4	4.4 ± 1.4	4.8 ± 1.3	*P* < 0.001	NS	NS
Set 2	6.6 ± 1.4^*^	5.8 ± 1.5^*^	5.8 ± 1.0^*^	6.2 ± 1.1^*^			
Set 3	7.6 ± 1.3^*#^	6.7 ± 1.4^*#^	7.1 ± 0.8^*#^	7.3 ± 0.8^*#^			
Set 4	8.3 ± 1.0^*^	7.8 ± 0.8^*#^	8.1 ± 1.2^*#^	8.1 ± 0.7^*#^			
Set 5	8.8 ± 1.0^*^	8.7 ± 1.0^*#^	8.8 ± 0.9^*#^	8.7 ± 0.7^*#^			
Difficulty with breathing							
Set 1	4.7 ± 1.0	4.6 ± 1.2	4.3 ± 1.4	4.3 ± 1.1	*P* < 0.001	NS	NS
Set 2	6.1 ± 1.2^*^	6.1 ± 1.2^*^	5.6 ± 1.1^*^	5.7 ± 1.2^*^			
Set 3	7.0 ± 1.4^*#^	7.0 ± 1.0^*#^	6.8 ± 0.8^*#^	6.9 ± 1.2^*#^			
Set 4	8.2 ± 1.0^*#^	8.1 ± 1.0^*#^	8.0 ± 1.0^*#^	7.9 ± 0.9^*#^			
Set 5	8.8 ± 1.0^*#^	8.8 ± 1.0^*#^	8.8 ± 1.0^*#^	8.9 ± 0.7^*#^			

Data are means ± SD. ^*^ *P* < 0.05 versus Set 1. ^#^ *P* < 0.05 versus previous set. No significant differences were found between conditions for all analyses. HB, hypoxia with BFR; HC, hypoxia with control BFR; NB, normoxia with BFR; NC, normoxia with control BFR.

**TABLE 3:** Lactate concentrations before and after repeated sprint exercise.

	NC	NB	HC	HB	Main effects		Interaction
					Time	Condition	
Blood lactate (mmol/l)							
Pre‐RSE	2.8 ± 1.1	2.6 ± 1.0	2.4 ± 0.7	3.7 ± 1.8	*P* < 0.001	NS	NS
Post‐RSE	14.2 ± 2.9^*^	13.2 ± 3.0^*^	14.6 ± 3.6^*^	13.7 ± 3.4^*^			

Data are means ± SD. ^*^ *P* < 0.05 versus Pre‐RSE. No significant differences were found between conditions for all analyses. HB, hypoxia with BFR; HC, hypoxia with control BFR; NB, normoxia with BFR; NC, normoxia with control BFR; RSE, repeated sprint exercise.

## 4 DISCUSSION

The present study investigated the effect of BFR administered during the rest periods of RSE training in a hypoxic environment. Our main finding was that RSE training under these conditions led to greater hypoxia within the local exercising muscle compared to the single intervention (NB and HC) or control conditions (NC) alone. Importantly, a combined intervention (HB) did not affect RSE workload or other associated RSE performance metrics. It follows that BFR administered during the rest periods of an RSE in hypoxic environmental conditions is an effective training modality for inducing potent local hypoxia in exercised muscles that does not compromise power output or workload (Table 1).

In the current study, neither the maximal nor the mean power output during a single set of 5 × 10 s RSE differed significantly between normoxic and hypoxic conditions. Similar to our findings, the initial phase of sprint performance was not reduced under a hypoxic condition during three sets of 5 × 5 s maximal pedalling (FiO_2_: 14.5%) (Goto et al., 2018), three sets of 5 × 6 s maximal cycling (FiO_2_: 14.6%) (Faiss et al., 2013), and a single set of 10 × 10 s maximal cycling (FiO_2_: 13.0%) (Smith & Billaut, 2010). During sprint exercise under hypoxia, anaerobic energy supply via the glycolytic pathway is augmented to compensate for the reduced aerobic energy supply (Billaut & Buchheit, 2013; Puype et al., 2013), which may explain why RSE performance was not compromised under the hypoxic conditions of our study. In contrast, others have shown that the ability to reproduce total mechanical work in subsequent sprints was impaired in hypoxic conditions (Smith & Billaut, 2010; Willis, Borrani et al., 2019). Although inconclusive, this discrepancy may be due to differences in RSE protocols such as the number of sets performed during the RSE (i.e., duration). For example, sprint performance under systemic hypoxia (13% O_2_) was comparable to that under normoxia in the early phase of RSE (Set 1–4); however, an obvious reduction in performance was found in the later phase of RSE (Set 5–10) (Smith & Billaut, 2010). From this perspective, the RSE performance in our protocol (single set of 5 × 10 s RSE) was preserved in acute hypoxia exposure.

In our study, SmO_2_ levels were lower during systemic hypoxia than during times of normoxia during both rest and sprint periods (Figure 3c,d). BFR lowered SmO_2_ even further under conditions of systemic hypoxia during the rest periods (Figure 3d). These data suggest that a combined intervention (BFR + systemic hypoxia) may result in severe local hypoxia in exercising muscles, potentially augmenting the benefit of the RSE training. During sprint periods, however, no additional reduction in SmO_2_ was detected in conditions of systemic hypoxia with BFR application (Figure 3c). This may be because BFR was only administered during RSE rest periods.

To date, most researchers have focused on the effect of BFR applied during periods of exercise (Peyrard et al., 2019; Valenzuela et al., 2019; Willis et al., 2018). During high‐intensity exercise, however, dynamic muscle contraction prevents the BFR (40–80% of AOP) cuff from occluding venous blood flow within exercising muscles (D. Kim et al., 2015; Patterson et al., 2019). Accordingly, researchers have commonly utilized BFR with low‐intensity exercises (Barcelos et al., 2015; Davids et al., 2021). Recently, it was shown that BFR with 130 mmHg of occlusion pressure administered during the rest periods of a sprint interval exercise (SIE), which consists of a long duration sprint (20–30 s) followed by several minutes of rest, could induce an increase in HIF‐1α mRNA expression within the exercising muscles (Taylor et al., 2016). This suggested that BFR elicits a potent metabolic stress even when applied to the rest periods of other training sessions, specifically high‐intensity exercise training such as RSE. Although we were not able to identify the underlying mechanisms implicated by these results at the molecular level, future studies may determine the metabolic factors within the working muscles that are triggered after BFR is applied during the rest periods of RSE under conditions of systemic hypoxia.

In the present study, BFR application significantly increased total‐Hb in a normoxia and hypoxia environment during both sprints and rest, respectively (Figure 3a,b). In BFR conditions (60% of AOP), venous blood flow is nearly abolished while arterial blood flow is partially allowed, thereby increasing local blood volume in the occluded limb (Willis et al., 2018). Along this line, a recent study by Willis, Peyrard et al. (2019) reported that total‐Hb was significantly higher in the BFR condition (45% of AOP) than in the non‐BFR state.

Also, total‐Hb was higher under systemic hypoxia than normoxia in non‐BFR conditions (NC vs. HC) during rest periods (Figure 3b). This is consistent with a previous study showing that total‐Hb levels are significantly higher during leg cycling (Faiss et al., 2013) and double poling exercises (Yamaguchi et al., 2019) during times of systemic hypoxia. The reduction in available O_2_ associated with systemic hypoxia promotes compensatory vasodilatation and increases blood flow within the exercised skeletal muscles (Casey & Joyner, 2012; Wilkins et al., 2008). Although the current study did not assess the dilatation of deep arteries during systemic hypoxia, the elevated total‐Hb levels suggests that hypoxia‐induced vasodilatation occurred. Together, the combination of BFR and systemic hypoxia (HB) increased total‐Hb even further (Figure 3b). However, total‐Hb is an indirect measure of blood volume by NIRS signals, and skin blood flow has been shown to significantly influence NIRS measures at rest and during exercise (Davis et al., 2006; Tew et al., 2010). Thus, the possibility cannot be ruled out that changes in skin blood flow would have an effect on total‐Hb in our study.

We measured surface EMG from the VL during the sprint periods of RSE to assess neuromuscular activity. The RMS of the EMG represents the amplitude of muscular activation, while the MF indicates the firing rate of the motor neurons of the muscle fibres (Fukuda et al., 2010). In the current study, both RMS and MF declined over the course of time, regardless of condition (Time effect: *P* < 0.0001 for both RMS and MF) (Figure 4a,b). Previous studies investigating neuromuscular activity during RSE reported steady reductions in motor unit activity due to peripheral metabolic disturbances (Mendez‐Villanueva et al., 2012), and it has been suggested that this indicates muscular fatigue (Billaut & Basset, 2007; S. Kim & Hurr, 2020; Mendez‐Villanueva et al., 2012, 2008). In this regard, our EMG data indicate that neuromuscular fatigue was induced over time during RSE training.

Interestingly, we observed a trend of elevated neuromuscular activity, as indexed by EMG RMS and MF, in response to BFR interventions, particularly under conditions of systemic hypoxia (HB) (Figure 4a,b). Several studies have suggested that the nervous system recruits all possible motor unit pools at their highest activation during supramaximal sprints (Krustrup et al., 2004). Given that no differences in perceived fatigue (Table 2) and blood lactate concentration (Table 3) were found between conditions following the RSE, we speculate that motor neuron activation during sprints may be increased to maintain muscular force as a compensatory mechanism when severe local hypoxia is induced (HB). Similar to our findings, increased neuromuscular activation was observed during exercise with BFR (60% of AOP) (Husmann et al., 2018), which is interpreted as an increased recruitment of type II muscle fibres (Pearson & Hussain, 2015). However, it cannot be ruled out that elevated EMG signals in HB might have been, at least in part, due to peripheral changes (i.e., a reduction in metabolic disturbances) rather than central neural drive.

### 4.1 Limitations

Our study is limited in some ways. Previous studies have applied individualized pressure for BFR application (∼50% AOP) (Valenzuela et al., 2019; Willis et al., 2018) to minimize inter‐individual variability that may create different physiological responses and exercise performance (Ilett et al., 2019). Considering dynamic changes in cardiovascular responses in unacclimatized healthy individuals during acute exposure to hypoxia (Theunissen et al., 2022), a constant cuff pressure (140 mmHg) was applied in our study. However, it should be noted that inter‐individual variability exists in the current study and our findings should be interpreted with caution.

We note the lack of women in our study. Females may experience higher elevations in absolute and relative blood flow during exercise (Parker et al., 2007), and be more sensitive to vascular changes that occur in response to arterial occlusion (Levenson et al., 2001) and reactive hyperaemia (Dankel et al., 2017). Although a previous study has shown that haemodynamic changes in response to resistance exercise are similar between the sexes (Mattocks et al., 2018), it remains unknown if sex differences would affect the results of this study.

In addition, we were unable to blind the participants to BFR application as participants could easily recognize the pressure of the BFR cuff. Moreover, while we attempted to blind participants to the hypoxic environment, they were able to recognize this as well. Thus, psychological effects (i.e., placebo effect) in regard to the examined interventions cannot be ruled out and caution is accordingly needed in interpreting the current data. Finally, RSE training is considered to be most suitable for highly trained athletic individuals (Spencer et al., 2005). Future studies are warranted to assess the acute or long‐term effect of the tested RSE training technique on professional athletes.

### 4.2 Conclusions and perspectives

In brief, RSE training that features BFR administered during rest periods under hypoxic environmental conditions would have the potential to improve the effectiveness of training. Severe local hypoxia within the exercised muscles can be induced by a combined intervention (BFR + systemic hypoxia) during RSE training without compromising the training workload or other performance associated with RSE. The power output that was maintained despite the severe local hypoxia might be, at least in part, due to elevated neuromuscular activity within the working muscles. Of course, future research will be needed to confirm that highly trained athletes would benefit from the tested RSE training technique.

## AUTHOR CONTRIBUTIONS

All experiments were conducted in the Integrative Exercise Laboratory at Jeonbuk National University. Study design, data analysis and data interpretation: Anjie Wang, R. Matthew Brothers and Chansol Hurr. Drafting: Anjie Wang. Editing: Anjie Wang, R. Matthew Brothers and Chansol Hurr. Data collection: Anjie Wang. All authors approved the final version of the manuscript, agree to be accountable for all aspects of the work, and will ensure that any questions concerning the accuracy or integrity of any part of this work are appropriately investigated and resolved. All persons designated as authors qualify for authorship, and all those who qualify for authorship are listed.

## CONFLICT OF INTEREST

The authors declare no conflicts of interest.

## Supporting information

Statistical Summary Document

## Data Availability

The data that support the findings of this study are available from the corresponding author upon reasonable request.
